# Association between nocturia and sleep issues, incorporating the impact of lifestyle habits perceived as promoting sleep in an internet survey

**DOI:** 10.1038/s41598-025-02587-7

**Published:** 2025-05-20

**Authors:** Haruna Nonaka, Shuhei Suzuki, Hiromitsu Negoro, Atsushi Ikeda, Ichiro Chihara, Shuya Kandori, Hiroyuki Nishiyama

**Affiliations:** 1 Department of Urology, Institute of Medicine, University of Tsukuba, 1-1-1 Tennodai, Tsukuba, Ibaraki, 305-8575 Japan; 2 Hitachi Social Cooperation Education Research Center, University of Tsukuba, 1-1-1 Tennodai, Tsukuba, Ibaraki, 305-8575 Japan; 3 Department of Urology, Hitachi General Hospital, 2-1-1 Jonan-cho, Hitachi, Ibaraki, 317-0077 Japan

**Keywords:** Age, Behavioral factors, Gender differences, Sleep satisfaction, Health care, Medical research, Urology

## Abstract

A mutual relationship between nocturia and sleep disturbances is assumed; however, evidence for these associations in the general population remains limited, particularly regarding sleep-promoting lifestyle habits. This cross-sectional internet study examined associations between nocturia, sleep issues, and lifestyle habits perceived as promoting sleep in 3,317 participants in July 2019. The prevalence of nocturia increased with age, while overall sleep satisfaction tended to improve with age. However, individuals with more frequent nocturnal voids reported lower sleep satisfaction. Sleep dissatisfaction was significantly correlated with nocturnal urinary frequency in both men (*r* = 0.16) and women (*r* = 0.18, *p* < 0.001), emphasizing the impact of nocturia on sleep quality. Furthermore, it revealed that nocturia was associated with various sleep issues. In men, mid-wakefulness (OR = 3.32, *p* < 0.001) and difficulty falling asleep (OR = 1.37, *p* < 0.050) were key factors, whereas in women, mid-wakefulness (OR = 11.2, *p* < 0.001) and shallow sleep (OR = 1.77, *p* = 0.010) were significant. Notably, it was found that lifestyle habits, such as drinking tea or alcohol, which can exacerbate nocturia and reduce sleep quality, are undertaken with intention of promoting good sleep. Conversely, good bedding (OR = 0.75, *p* = 0.010) was associated with fewer nocturnal voids. These findings highlight the complex interplay between nocturia, sleep issues, and lifestyle behaviors, providing valuable insights for addressing these interconnected issues.

**Supplementary Information:**

The online version contains supplementary material available at 10.1038/s41598-025-02587-7.

## Introduction

Nocturia is characterized by the need to wake up to pass urine during the main sleep period, with each urination followed by sleep or the intention to sleep^1^. According to the International Consultation on Incontinence Research Society 2018 definition, waking two or more times per night to urinate is considered clinically relevant^2^. Nocturia is one of the most common and bothersome lower urinary tract symptoms^1,3^, and the incidence of nocturia increases markedly with age in individuals. More than 60% of people aged 60 and older experience this symptom^4^. Frequent nighttime awakenings to urinate not only reduce quality of life but also increase the risk of falls and fractures^5,6^. Moreover, nocturia has been associated with systemic health problems, including cardiovascular disease, metabolic abnormalities, neurological conditions, and malignancies^7,8^. A systematic review and a subsequent paper indicated that nocturia may increase the risk of death by approximately 1.3-fold, with higher frequencies of nighttime voiding correlating with greater risk^9,10^. These findings underscore nocturia as an important clinical issue requiring appropriate management.

Nocturia can be caused by three factors: polyuria/nocturnal polyuria, decreased bladder capacity, and sleep disturbance. However, the underlying mechanisms are diverse and complex, making satisfactory treatment challenging^6^. In particular, the bidirectional relationship between nocturia and sleep disturbances adds further complexity^11^.

Sleep issues have a significant negative impact on health, and the economic loss due to sleep deprivation is estimated to be tens of billions of dollars per year in the United States^12^. One of the most common reasons for interrupted sleep in the general adult population is nocturia, and nocturia-induced sleep issues have been associated with clinical condition including day-time fatigue, depression, increased hip fracture risk and a significant reduction in general health-related quality of life^5,13–15^. Furthermore, a Japanese study examined the relationship between sleep issues and urinary conditions, and found sleep issues were significantly associated with the prevalence and worsening of urinary conditions^16^. These findings suggest a mutual, potentially cyclical relationship between sleep disturbances and nocturia.

However, evidence demonstrating the relationship between nocturia and sleep issues among the general population remains limited^17–19^. Particularly, there is limited understanding of sleep-promoting lifestyle habits that the general population engages in, as well as attempts to manage sleep issues^20–22^. Appropriate understanding and implementation of good habits for nocturia and sleep quality may help avoid excessive or ineffective pharmacotherapy. Therefore, in this study, we conducted an Internet-based survey in Japan to investigate the relationship between nocturia and sleep issues, as well as to examine the association between nocturia and lifestyle habits aimed at promoting good sleep.

## Results

### Participants’ characteristics

Participants’ characteristics are presented in Table 1. A total of 3,317 were included in the analysis. The median age was 55 years (quartile range: 48–63,), and the male to female ratio was generally equal (51.5% male vs. 48.5% female). Furthermore, 75.4% of the participants were married (2,501 individuals). The distribution of participants by residential region was as follows: 184 (5.5%) in Hokkaido, 195 (5.9%) in Tohoku, 1,293 (39.0%) in Kanto, 492 (14.8%) in Chubu, 688 (20.1%) in Kinki, 134 (4.0%) in Chugoku, 90 (2.7%) in Shikoku, and 241 (7.3%) in Kyushu (Fig. S1).

**Table 1:** Characteristics of the participants.

Number of participants	3317
Sex	
Men (n, %)	1707 (51.5)
Women (n, %)	1610 (48.5)
Median Age, years (quartile, range)	55 (48–63, 40–75)
Married (n, %)	2501 (75.4)
Region	
Hokkaido (n, %)	184 (5.5)
Tohoku (n, %)	195 (5.9)
Kanto (n, %)	1293 (39.0)
Chubu (n, %)	492 (14.8)
Kinki (n, %)	688 (20.1)
Chugoku (n, %)	134 (4.0)
Shikoku (n, %)	90 (2.7)
Kyushu (n, %)	241 (7.3)

### Sleep issues and nocturia

**Fig. 1:**
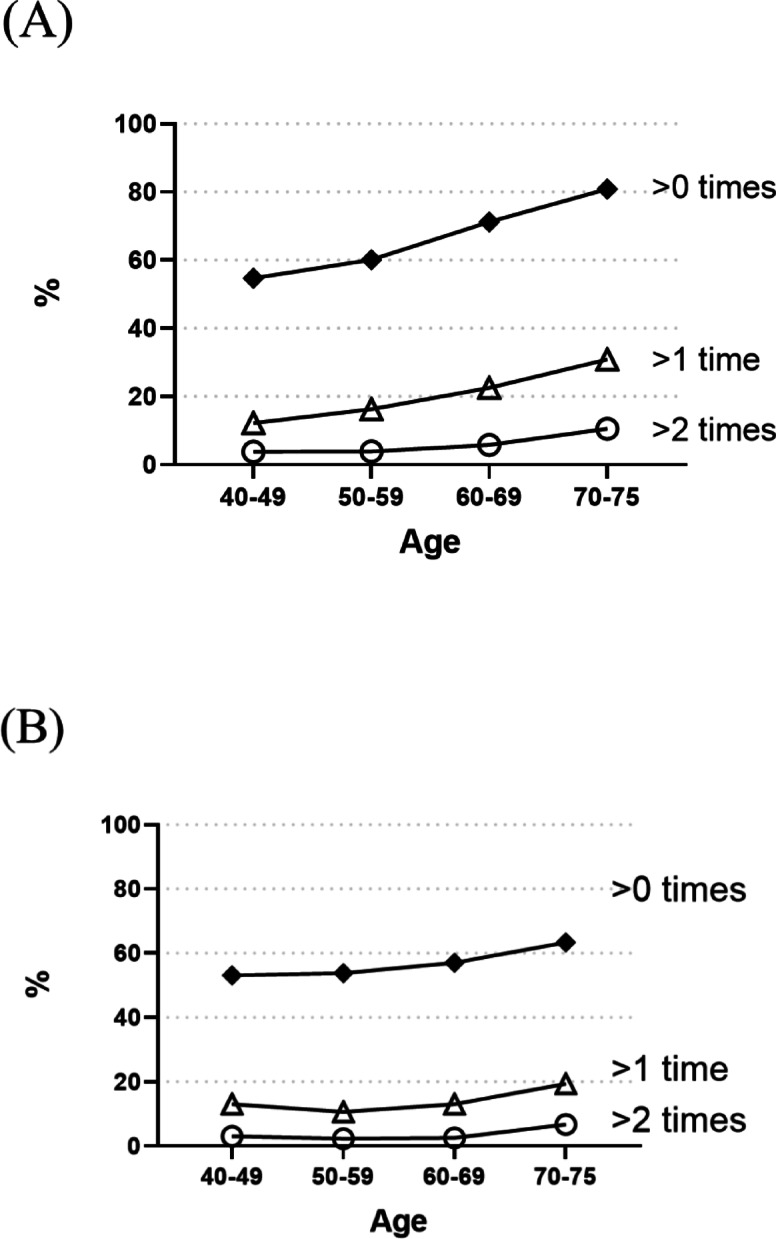
Prevalence of nocturia by age and gender. (**A**) Men. (**B**) Women.

**Fig. 2:**
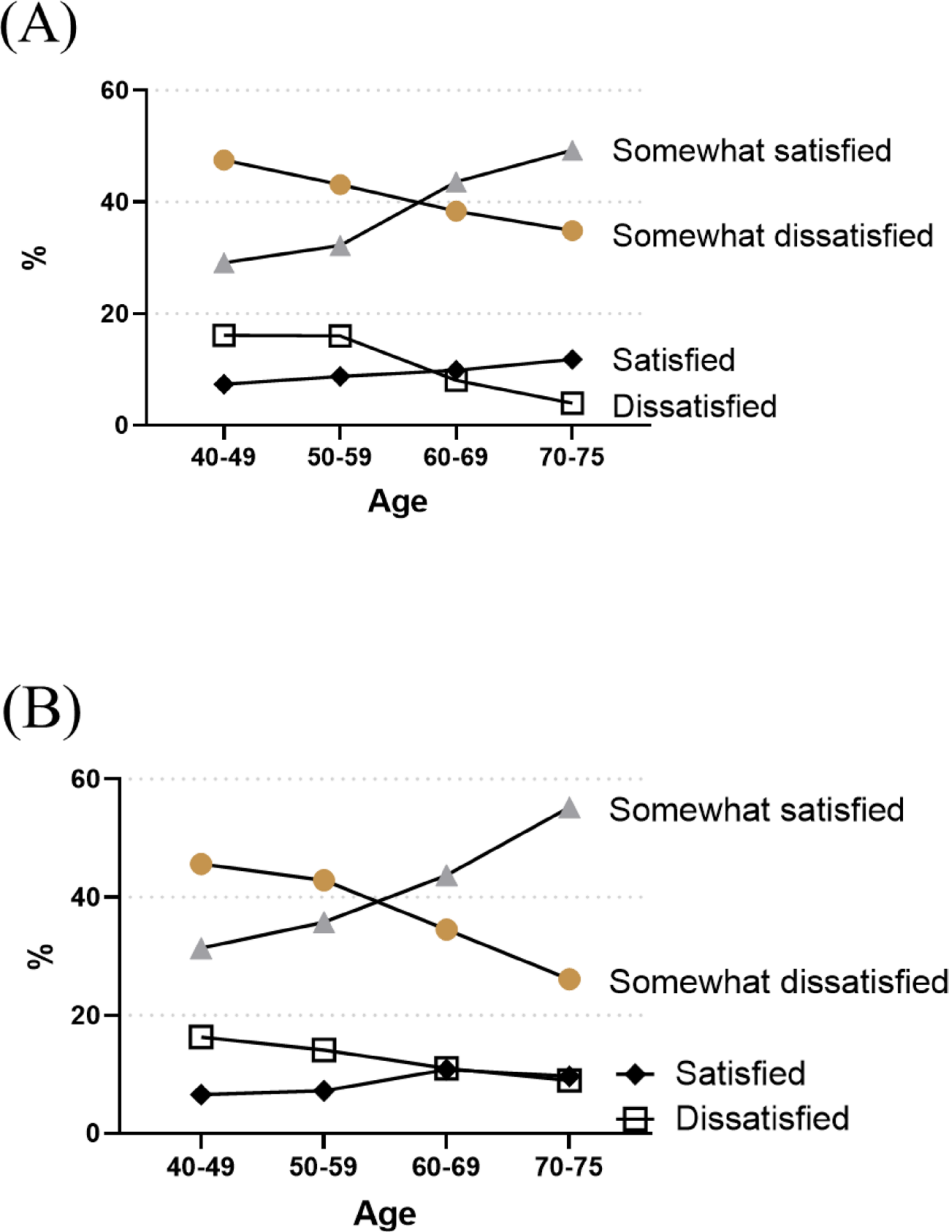
Prevalence of sleep dissatisfaction by age and gender. (**A**) Men. (**B**) Women.

**Fig. 3:**
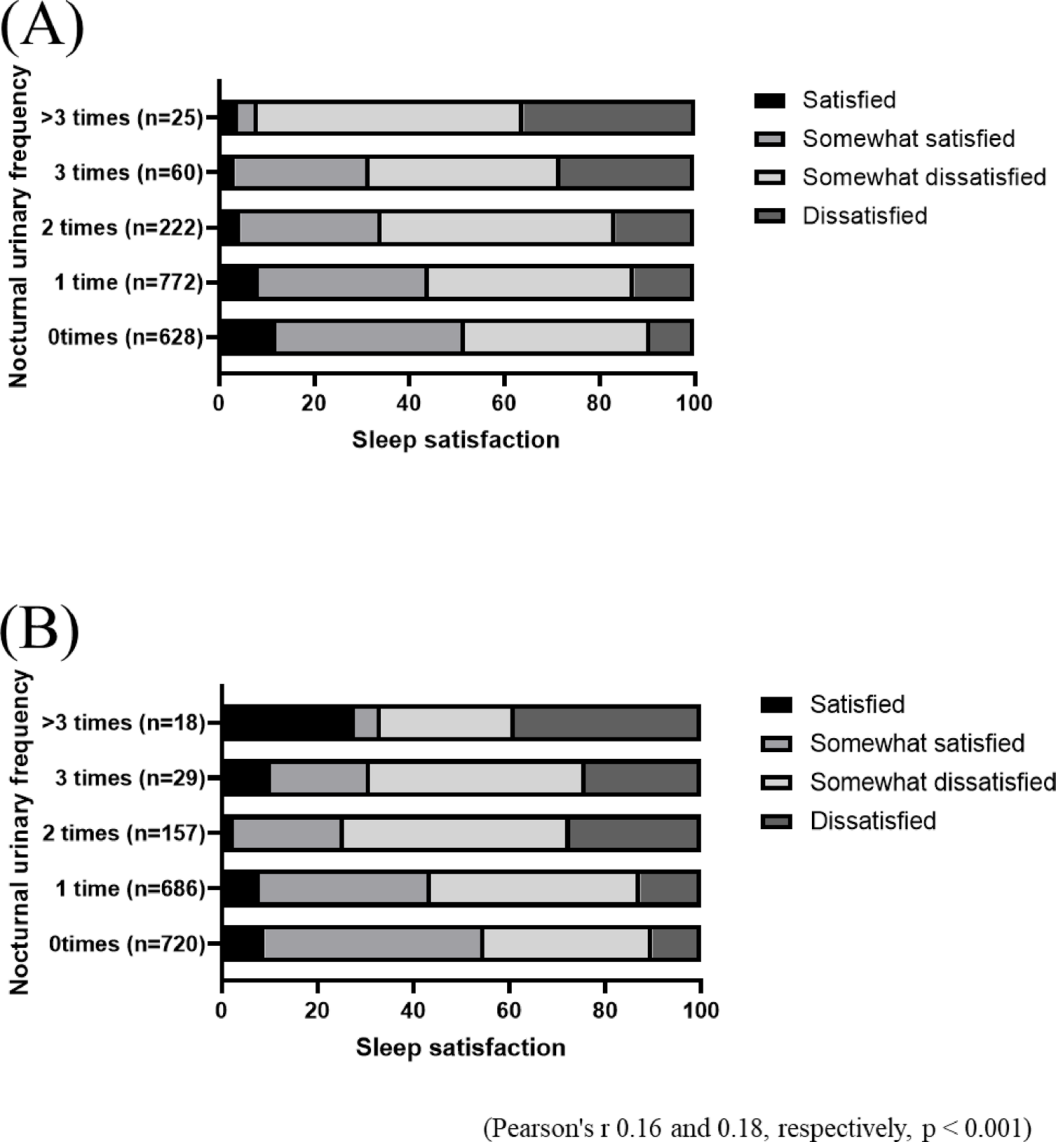
Relations of sleep dissatisfaction and nocturia in men (**A**) and women (**B**).

The number of participants with nocturnal urinary frequency was 1,348 (41.0%) for 0, 1458 (44.0%) for 1, 379 (11.0%) for 2, 89 (3.0%) for 3, and 43 (1.0%) for more than 3. Figure 1 shows the prevalence of nocturia by age and gender. The prevalence of nocturia increased with age, regardless of gender. Especially in the 70–75 age group, the proportion of participants with nocturia more than once was higher in both men (30.9%) and women (19.4%). Figure 2 shows the prevalence of sleep dissatisfaction by age and gender. In contrast to the prevalence of nocturia, the prevalence of dissatisfaction or somewhat dissatisfaction with sleep decreased with age in both men (63.6%, 59.1%, 46.5%, and 38.8%) and women (62.0%, 57.1%, 45.5%, and 35.1%). However, when the sleep satisfaction was evaluated by stratifying according to nocturnal urinary frequency, there was a tendency for sleep satisfaction to worsen as the nocturnal urinary frequency increased. A significant correlation was found in both men and women (Pearson’s *r* = 0.16 and 0.18, respectively, *p* < 0.001) (Fig. 3). When analyzed by age, the percentage of participants who were not satisfied with their sleep was significantly higher in the younger adult group (Fisher’s Exact Test, *p* < 0.050). Participants who were not satisfied with their sleep were more prevalent in the group with high nocturnal urinary frequency, and this was consistent for both the younger (< 60 years) and older adults (≥ 60 years) groups (Fisher’s Exact Test, *p* < 0.050 for each) (Fig. S2).

The sleep issues correlated with nocturnal urinary frequency are shown in Table S1. Nocturia was most strongly associated with mid-wakefulness and was weakly associated with various other sleep issues. In a logistic regression analysis adjusted for age, nocturia (≥ 2 vs. <2) was significantly associated with the majority of sleep issues. A multivariable analysis of the correlation between all sleep issues and nocturia (≥ 2 vs. <2), adjusted by age and sleep issues, showed significant associations with mid-wakefulness (OR 3.32, *p* < 0.001) and difficulty falling asleep (OR 1.37, *p* < 0.050) in men, and mid-wakefulness (OR 11.2, *p* < 0.001), shallow sleep (OR 1.77 *p* = 0.010) in women (Table 2).

**Table 2:** Sleep issues correlated with nocturia in men (A) and women (B).

(A)	Nocturnal urinary frequency		Adjusted odds ratio	
	≥ 2 (*n* = 307)	< 2 (*n* = 1400)		
	n (%)	n (%)	OR (95% CI)	*P* value
Mid-wakefulness	256 (83.4)	776 (55.4)	3.32 (2.32–4.75)	< 0.001
Difficulty falling asleep	115 (37.5)	355 (35.4)	1.37 (1.00-1.88)	< 0.050

(B)	Nocturnal urinary frequency		Adjusted odds ratio	
	≥ 2 (*n* = 204)	< 2 (*n* = 1406)		
	n (%)	n (%)	OR (95% CI)	*P* value
Mid-wakefulness	194 (95.1)	769 (54.7)	11.2 (5.6–21.2)	< 0.001
Shallow sleep	166 (81.4)	689 (49.0)	1.77 (1.13–2.76)	0.010

*OR* odds ratio, *CI* confidence interval.

### Relationship between attempts to achieve good sleep and nocturia

The correlation between attempts to achieve good sleep and nocturnal urinary frequency is shown in Table S2. Nocturnal urinary frequency was significantly associated with most lifestyle habits according to Spearman’s rank correlation coefficient test, except for taking a bath, dimming room lights, and using good bedding. Table S3 summarizes the frequency of ten lifestyle habits perceived as promoting sleep across four groups: individuals with nocturia and unsatisfied sleep, with nocturia and satisfied sleep, without nocturia and with unsatisfied sleep, and without nocturia and with satisfied sleep. Dimming room lights and using the bathroom before bedtime were common across all groups (77.6–80.5%). Additionally, individuals with nocturia, regardless of sleep satisfaction, appeared more likely to drink tea or other sleep-promoting beverages and to consume alcohol (19.5–21.7% vs. 31.5–40.1%). Multivariable analysis for correlation of nocturia (≥ 2 vs. <2) and all lifestyle habits adjusted by age, lifestyle habits, and sleep satisfaction, identified factors that were significantly associated with the frequency of nocturia. Drinking tea or other beverages that facilitate sleep (OR 1.70, *p* < 0.001), consuming alcohol (OR 1.59, *p* < 0.001), and limiting water intake (OR 1.39, *p* = 0.006) were significantly associated with a higher frequency of nocturia, while using good bedding (OR 0.75, *p* = 0.010) was the only significant factor associated with lower frequency of nocturia (Table 3).

**Table 3:** Association between sleep-promoting lifestyle habits and nocturia.

	Nocturnal urinary frequency		Adjusted odds ratio	
	≥ 2 (*n* = 511)	< 2 (*n* = 2806)		
	n (%)	n (%)	OR (95% CI)	*P* value
Drinking tea or other beverages that facilitate sleep	103 (20.1)	351 (12.5)	1.70 (1.30–2.21)	< 0.001
Consuming alcohol	174 (34.1)	683 (24.3)	1.59 (1.28–1.96)	< 0.001
Limiting water intake	144 (28.2)	572 (20.4)	1.39 (1.10–1.75)	0.006
Using good bedding	181 (35.4)	1046 (37.3)	0.75 (0.60–0.94)	0.010

*OR* odds ratio, *CI* confidence interval.

## Discussion

This study revealed that nocturia is correlated with various sleep issues, including mid-wakefulness, difficulty falling asleep, and shallow sleep in the Japanese general population using an Internet survey. It also identified gender- and age-related characteristics in the relationship between nocturia and sleep issues. This study also highlighted that while some sleep-promoting habits are appropriately recognized, others may need to be reconsidered in the context of nocturia.

Regarding aging, this survey showed that the prevalence of nocturia increases with age, while the condition of sleep dissatisfaction decreases. This may be because individuals who have reached retirement age are relieved of work-related stress, and as their domestic responsibilities, such as childcare, gradually decrease, they are able to get the sleep they need^23,24^. However, among older adults (≥ 60 years), individuals with fewer nocturnal urinary frequency tend to be more satisfied with their sleep, whereas those with a higher frequency of nocturia tend to have lower overall sleep satisfaction. In other words, although sleep satisfaction generally increases with age, severe nocturia may interfere with it. An observational cross-sectional study of patients admitted to a urology department reported that sleep disorders were more common in patients with LUTS^25^, and another cohort study showed that men with nocturia were more likely to experience excessive daytime sleepiness^26^. These reports are consistent with our findings. Thus, there is a possibility that a significant number of patients with sleep issues may also require treatment for nocturia.

The same tendency was seen in younger adults (< 60 years), where a high percentage of participants with high nocturnal urinary frequency with dissatisfied with their sleep. However regardless of the nocturnal urinary frequency, the proportion of younger adults dissatisfied with their sleep was significantly higher. This suggests that factors other than nocturia contribute to sleep dissatisfaction in younger adults. Previous reports have identified several important risk factors for sleep issues, including obesity, unemployment, lack of a regular exercise routine, and mental stress^27^. Given that age and gender differences have also been reported, it is possible that factors other than nocturia play a major factor in sleep issues among younger adults.

Next, we examined the relationship between sleep issues and nocturia and found that mid-wakefulness was significantly correlated with nocturia in both genders. In addition, difficulty falling asleep was significantly associated with nocturia in men, whereas shallow sleep was significantly associated with nocturia in women. Although nocturia is a direct cause of mid-wakefulness, previous reports have suggested that the relationship between sleep issues and nocturia varies by gender^28,29^. As men age, they are more likely to develop urological diseases (such as benign prostatic hyperplasia), which are often accompanied by frequent nighttime urination. Exposure to light during nighttime urinations increases sympathetic nervous system activity and suppresses melatonin secretion. Previous studies have shown that melatonin not only promotes sleep and induces fatigue-like states but also ultimately leads to increased bladder capacity and decreased urine volume^6,30^. A reduced secretion of melatonin can make it more difficult to fall back asleep and may also affect the progression of nocturia. On the other hand, nocturia may also be indirectly associated with difficulty falling asleep. One possible explanation is that individuals who have experienced frequent nocturnal awakenings due to nocturia may develop anxiety or anticipatory arousal at bedtime, fearing repeated awakenings during the night. This may contribute to difficulty initiating sleep^31^. Conversely, it is also plausible that difficulty falling asleep itself contributes to increased nocturnal urine production. Prolonged wakefulness before sleep might reduce the secretion of antidiuretic hormone (ADH), leading to increased nocturnal urine volume and frequency^32^. In this sense, the relationship between nocturia and difficulty falling asleep may be bidirectional, and further studies are warranted to clarify the causal pathways. According to Kim et al., the age-related decline in sex hormones occurs more rapidly in women than in men^33^. These hormonal changes make women more prone to shallow sleep in middle age, which can lead to more frequent nocturia due to increased arousal^34^. Given these significant gender differences in sleep issues and nocturia, it is important to investigate the underlying causes separately for each gender and to implement proactive interventions for sleep issues associated with nocturia.

Nocturia and sleep issues may be improved by lifestyle modifications, which should be implemented prior to pharmacotherapy. Examples of beneficial lifestyle habits include minimizing fluid intake (especially caffeine and alcohol) at least 2 h before bedtime, limiting total fluid consumption to less than 2 L/day, emptying the bladder before going to bed, ensuring barrier-free access to a toilet, increasing exercise, reducing dietary salt intake, avoiding smoking, and achieving weight loss in overweight individuals^17,19^. Since beverages like coffee, which contain high levels of caffeine, can affect circadian rhythm and have diuretic effects, they should be avoided before bedtime^18^.

In this survey, we found that “Drinking tea or other sleep-inducing drinks” and “Drinking alcohol” were significantly more common among individuals with nocturia, regardless of gender. Notably, these habits were undertaken with the intention of promoting good sleep. However, consuming fluids before bedtime is unsuitable for those with nocturia, and drinking alcohol can adversely affect not only nocturia but also sleep quality. These findings provide valuable insights into the type of education that should be offered to the general population with sleep disorders and nocturia. In addition, “Not drinking too much water” was also significantly more common among those with nocturia. It is generally accepted that limiting fluid intake is an effective countermeasure for improving nocturia, and in some cases, physicians may advise patients to reduce their fluid consumption. This is thought to be the result of individuals with nocturia being aware that limiting fluid intake may help alleviate their symptoms and consciously making an effort to do so. This approach is generally appropriate for individuals whose nocturia is primally caused polyuria/nocturnal polyuria. However, indiscriminate fluid restriction carries the risk of heat-related illnesses, such as heatstroke. Therefore, it is essential to provide guidance on maintaining an appropriate level of fluid intake based on urine output (20–30 mL/kg body weight/day)^35^.A higher proportion of individuals in the group with nocturia and satisfied sleep reported engaging in moderate daytime exercise compared to those with unsatisfied sleep. Although no causal relationship can be inferred from these findings, this observation may suggest that certain daytime lifestyle factors, such as exercise, are associated with perceived sleep satisfaction in individuals with nocturia.

A multivariable analysis examining the correlation between nocturia (≥ 2 vs. <2 episodes) and various lifestyle habits, adjusted by age, lifestyle habits, and sleep satisfaction, suggested that using good bedding may be associated with a reduced risk of nocturia. In a previous study evaluating the effects of bedding materials on sleep quality, it was found that sleeping with a high rebound mattress topper induced a marked decline in core body temperature during the initial phase of nocturnal sleep, and this decline was associated with an increase in deep sleep. Furthermore, the study suggested that reduced muscle activity required for rolling over is another factor that improves sleep quality^36^. In addition, previous literature in sleep medicine suggests that bedding and sleepwear can influence sleep quality by affecting thermal comfort. For example, a systematic review by Xinzhu Li et al. reported that optimal bedding varies depending on the population and environmental conditions^37^. Wool bedding and sleepwear may promote faster sleep onset and deeper sleep in cooler conditions, especially among older individuals and those with sleep difficulties. In contrast, cotton sleepwear may be more suitable for healthy young adults under moderate temperatures. While our study cannot determine causality, nor specify the types of bedding used, it is possible that using bedding appropriate to individual needs and environmental conditions may contribute to improved sleep quality, and thereby indirectly help alleviate nocturia.

A strength of the present study was its large population size, which enabled age-stratified and sex-separated analyses. These analyses provided a clear understanding of the relationships among nocturia, sleep issues and lifestyle habits, and suggested that taking effective countermeasures tailored to each sleep issue may help to improve nocturia. However, several limitations must be acknowledged. First, there is a possibility of selection bias among survey participants. This survey was limited to individuals with internet access and those who participated in online surveys, many of whom may have had a particular interest in sleep issues. Nevertheless, the distribution of the participants across regions closely matched the national population ratio (Chi-square 2.121, df 7, *p* = 0.950) (Fig. S1). Although Internet surveys are highly anonymous and adherence to self-reported behaviors may be uncertain, large-scale nationwide epidemiological surveys have already been successfully conducted both in Japan and other countries^38,39^. We lacked clinical information on background factors for participants. The causes of nocturia are diverse, and lower urinary tract symptoms such as benign prostatic hyperplasia and overactive bladder, as well as internal diseases such as heart failure and diabetes, have a significant impact on the frequency of urination. In addition, drugs that have a diuretic effect, antipsychotic drugs, and steroids that affect sleep and wakefulness have a significant impact on nocturia and the degree of sleep and wakefulness. Further research that includes background factors such as these underlying diseases and medications would strengthen the present findings. Finally, as this is a cross-sectional study, it is difficult to determine the directionality of the relationship between nocturia and sleep issues. In the future, prospective cohort studies or interventional trials would be valuable for further advancing our understanding of this relationship.

In summary, the severity of nocturia was significantly correlated with lower sleep satisfaction and various sleep issues. Our findings also suggest that some individuals with nocturia may have misconceptions about which lifestyle habits promote good sleep. These insights could prove useful in guiding sleep hygiene modifications and in developing educational strategies for individuals with nocturia and sleep issues.

## Methods

### Study design and sample

In July 2019, we conducted a cross-sectional internet survey in collaboration with Intelligence Value, Inc. to examine the relationship between nocturia and lifestyle habits perceived as promoting sleep. Target participants were aged 40–75 years registered in JustSytems Corporation.

### Questionnaire

Participants answered six screening and general demographic questions covering sex, age, region of residence, employment status, marital status, and family structure. In addition, the questionnaire included items on sleep satisfaction, sleep issues, and nocturia. Sleep-related questions comprised one item on overall sleep satisfaction and ten items addressing specific sleep issues: Mid-wakefulness, Shallow sleep, Difficulty falling asleep again after waking, Difficulty falling asleep, Feeling very sleepy during the day, Poor wake up, Difficulty leaving the bed, Insufficient sleeping time, Sleeping longer on weekends than on weekdays, and Not going to bed at a regular time. The frequency of urination from bedtime to morning awakening was also assessed.

Lifestyle habits for promoting good sleep were assessed using a series of items addressing behaviors such as Taking a bath; Dimming room lights; Refraining from looking at a cell phone or smartphone after getting into bed; Going to bed at a fixed time as much as possible; Engaging in moderate exercise during the day; Limiting water intake; Using the bathroom as much as possible before bedtime; Drinking tea or other beverages that facilitate sleep; Consuming alcohol; and Using good bedding. Each item was rated on a four-point Likert-type scale with the following response options: Very true, Somewhat true, Somewhat untrue, and Very untrue with an additional ‘Unknown’ option. A total of 4,272 individuals responded to the questionnaire; among these, 3,317 respondents who provided complete responses—i.e., no items answered as ‘Unknown’ or left blank—were included in the analysis.

### Statistical analyses

Participant characteristics and scores were analyzed using Fisher’s exact test and the Chi-square test. Correlations were analyzed using Pearson’s and Spearman’s correlation coefficient tests. Frequencies of attempts to achieve good sleep across three groups stratified by nocturia and sleep satisfaction were analyzed using the chi-square test. In the multivariable analysis for correlation of nocturia and all lifestyle habits, age and sleep satisfaction were used as covariates. Odds ratios were calculated using logistic regression analysis adjusted for age, and a p-value of < 0.050 was considered statistically significant. All analyses were performed using Graph Pad Prism software (version 10.2.2; GraphPad Software, La Jolla, CA, USA). The map of Japan shown in Figure S3 was created using Strat Point, a web-based platform (https://www.start-point.net/) that permits free use without copyright restrictions.

### Ethical approval

Data were collected anonymously and provided in a completely unlinked and anonymized format, making it impossible to identify individual participants. Therefore, the right to withdraw did not apply. Participation in the study was voluntary, and participants were informed that submitting their responses implied consent for data use. The study was approved by the Ethics Committee of the University of Tsukuba (approval number: 1570), ensuring compliance with ethical standards, protection of participants, and safe handling of data. This study was conducted in accordance with the ethical guidelines for medical and health research involving human subjects as enforced by the Japanese Ministry of Health, Labour, and Welfare, and the Declaration of Helsinki.

## Electronic supplementary material

Below is the link to the electronic supplementary material.

Supplementary Material 1

## Data Availability

The datasets generated and/or analyzed during the current study are available from the corresponding author upon reasonable request.
