# Supplementary material for: Association between nocturia and sleep issues, incorporating the impact of lifestyle habits perceived as promoting sleep in an internet survey
Source: Sci Rep. 2025 May 20;15:17508. doi: 10.1038/s41598-025-02587-7 (PMC12092793; doi:10.1038/s41598-025-02587-7)
Supplement: Supplementary file 1 — Supplementary Material 1 [file 41598_2025_2587_MOESM1_ESM.docx]

***Supplementary material***

**Table S1** **Sleep problems correlated with nocturnal urination frequency in men and women.**

|  | Men | |  | Women | |
| --- | --- | --- | --- | --- | --- |
|  | Rank correlation coefficient | P value |  | Rank correlation coefficient | P value |
| Sleep satisfaction | -0.162 | <0.001 |  | -0.176 | <0.001 |
| Sleep issues |  |  |  |  |  |
| Mid-wakefulness | 0.47 | <.0001 |  | 0.47 | <0.001 |
| Shallow sleep | 0.24 | <0.001 |  | 0.29 | <0.001 |
| Difficulty falling asleep again | 0.17 | <0.001 |  | 0.25 | <0.001 |
| Difficulty falling asleep | 0.19 | <0.001 |  | 0.21 | <0.001 |
| Feeling very sleepy during the day | 0.09 | 0.001 |  | 0.13 | <0.001 |
| Poor wake up | 0.04 | n.s. |  | 0.13 | <0.001 |
| Difficulty leaving the bed | 0.03 | n.s. |  | 0.1 | <0.001 |
| Insufficient sleeping time | -0.02 | n.s. |  | 0.05 | 0.052 |
| Sleep longer on weekends than on weekdays | -0.02 | n.s. |  | 0.01 | n.s. |
| Not going to bed at a regular time | -0.01 | n.s. |  | 0.02 | n.s. |

**Table S2** **Correlation of attempts to good sleep and nocturnal urinary frequency.**

|  | **Spearman’s rho** | **P value** |
| --- | --- | --- |
| **Consuming alcohol** | -0.1247 | <0.001 |
| **Drinking tea or other beverages that facilitate sleep** | -0.0813 | <0.001 |
| **Using the bathroom before bedtime** | -0.0608 | <0.001 |
| **Going to bed at a fixed time as much as possible** | -0.0569 | <0.001 |
| **Limiting water intake** | -0.0562 | <0.010 |
| **Engaging in moderate exercise during the day** | -0.0543 | <0.010 |
| **Refraining from looking at a smartphone after getting into bed** | -0.0429 | 0.014 |
| Taking a bath | -0.0256 | 0.141 |
| Using good bedding | -0.0253 | 0.145 |
| Dimming room lights | -0.0237 | 0.174 |

**Table S3.** **Frequencies of attempts to achieve good sleep across groups stratified by nocturia and sleep satisfaction.**

|  | Nocturnal urinary frequency | | | | | |  |
| --- | --- | --- | --- | --- | --- | --- | --- |
|  | ≥2 | |  | | <2 | |  |
|  | Unsatisfied sleep | Satisfied sleep |  | Unsatisfied sleep | | Satisfied sleep |  |
|  | n (%) | n (%) |  | n (%) | | n (%) |  |
| Taking a bath | 208 (57.9) | 93 (61.2) |  | 776 (53.6) | | 788 (58.0) |  |
| Dimming room lights | 282 (78.6) | 118 (77.6) |  | 1165 (80.5) | | 1076 (79.2) |  |
| Refraining from looking at a cell phone or smartphone after getting into bed | 175 (48.7) | 83 (54.6) |  | 658 (45.5) | | 699 (51.4) |  |
| Going to bed at a fixed time as much as possible | 209 (58.2) | 104 (68.4) |  | 789 (54.5) | | 898 (66.1) |  |
| Engaging in moderate exercise during the day | 142 (39.6) | 74 (48.7) |  | 436 (30.1) | | 564 (41.5) |  |
| Limiting water intake | 102 (28.4) | 42 (27.6) |  | 294 (20.3) | | 278 (20.5) |  |
| Using the bathroom as much as possible before bedtime | 305 (85.0) | 124 (81.6) |  | 1237 (85.5) | | 1129 (83.1) |  |
| Drinking tea or other beverages that facilitate sleep | 70 (19.5) | 33 (21.7) |  | 178 (12.3) | | 173 (12.7) |  |
| Consuming alcohol | 113 (31.5) | 61 (40.1) |  | 350 (24.2) | | 333 (24.5) |  |
| Using good bedding | 124 (34.5) | 57 (37.5) |  | 484 (33.4) | | 562 (41.4) |  |

**Fig. S1.** **Comparison of the Regional Percentages in this survey (A) to the in Japan population (B).**


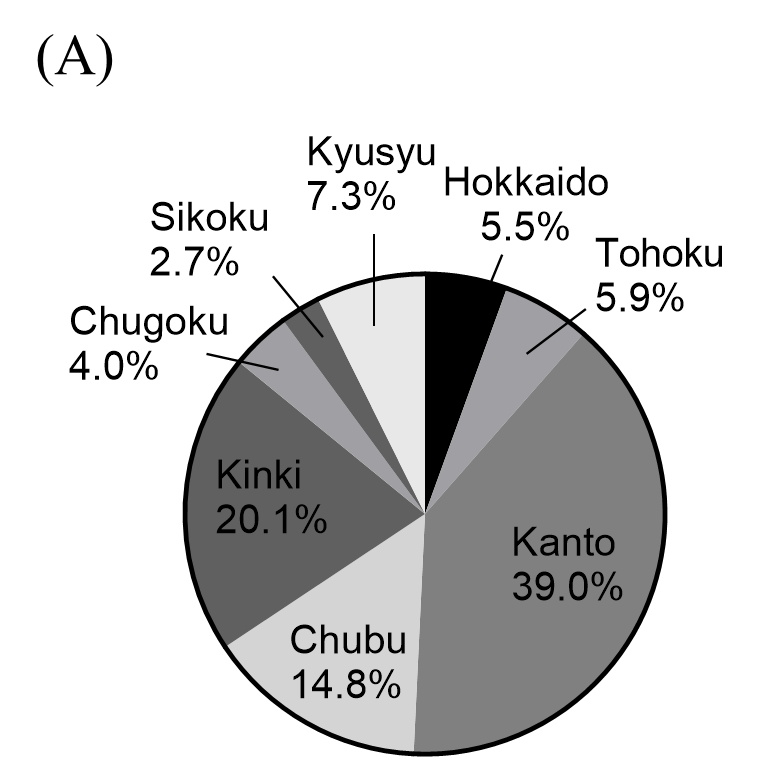


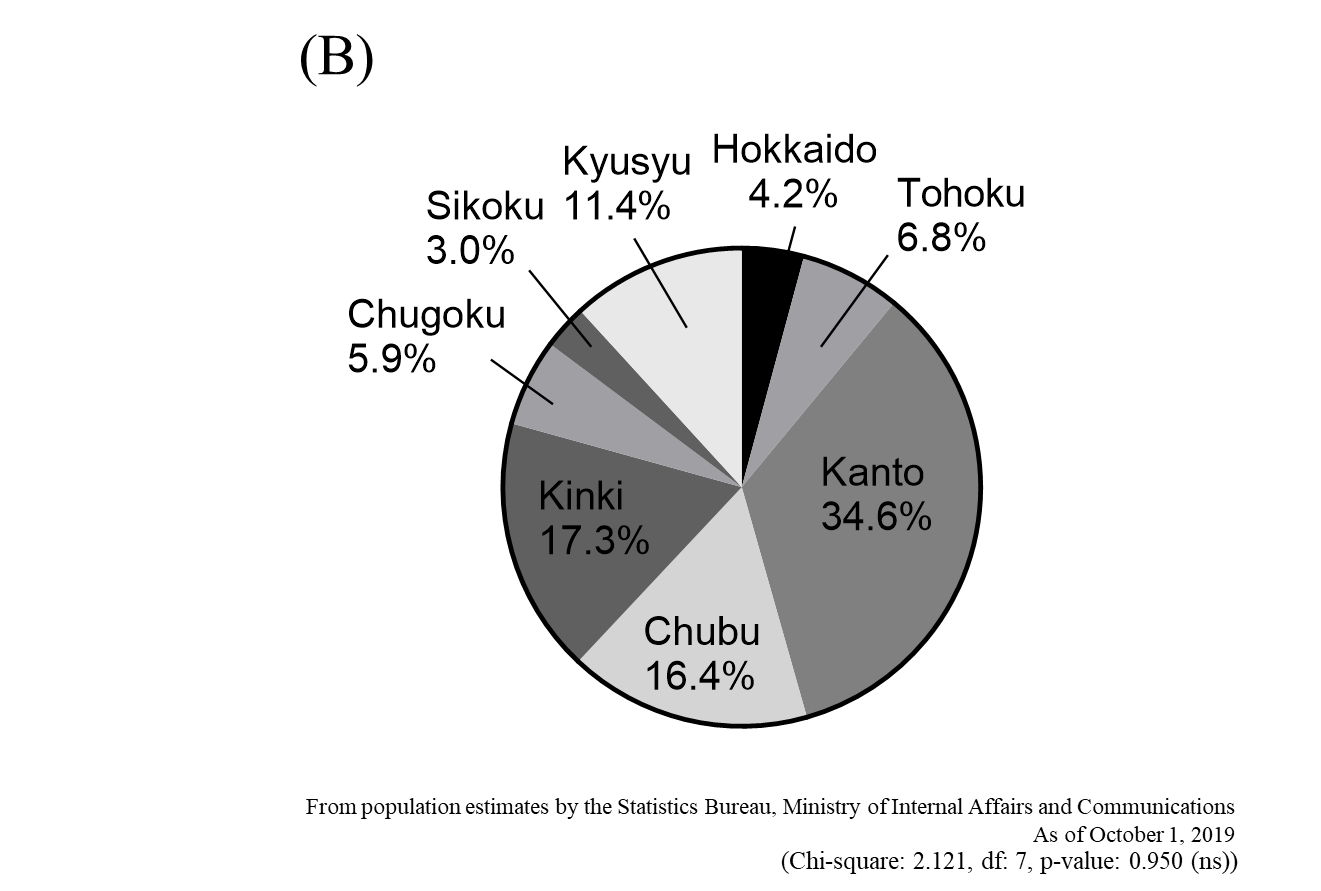


**Fig. S2.** **Relations of sleep satisfaction and nocturia in older (A) and younger adults (B).**

**
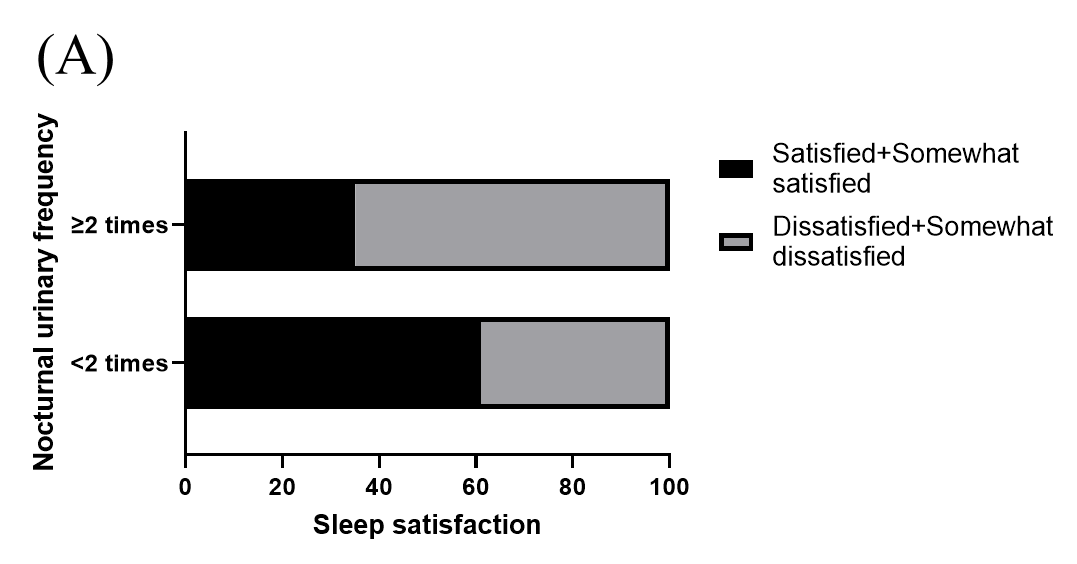
**

**
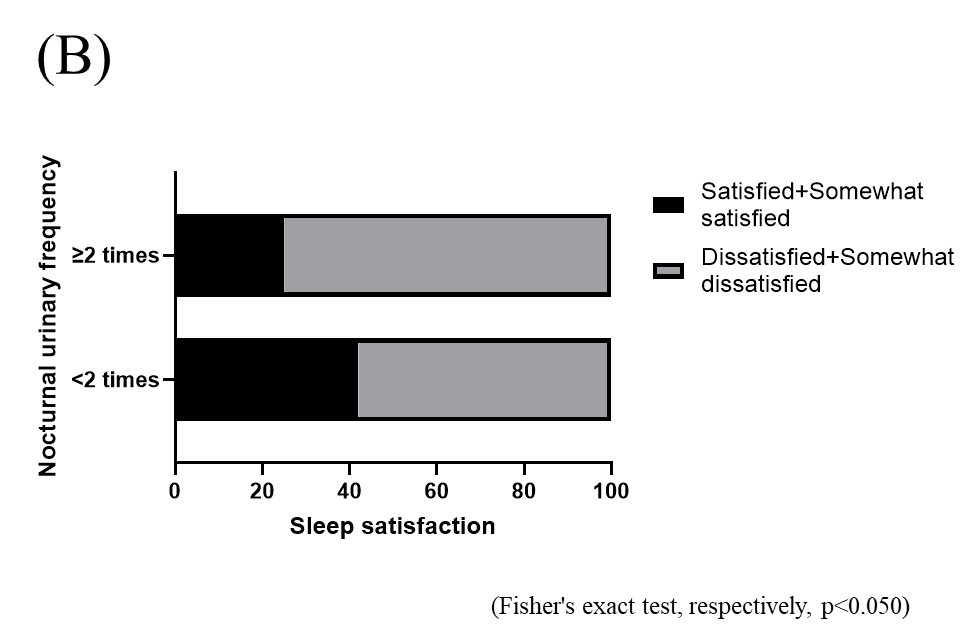
**

**Fig. S3. Map of Japan divided into seven regions (Hokkaido, Tohoku, Kanto, Chubu, Kinki, Chugoku, Shikoku, and Kyushu), color-coded according to the residential areas of participants included in this study.**

**
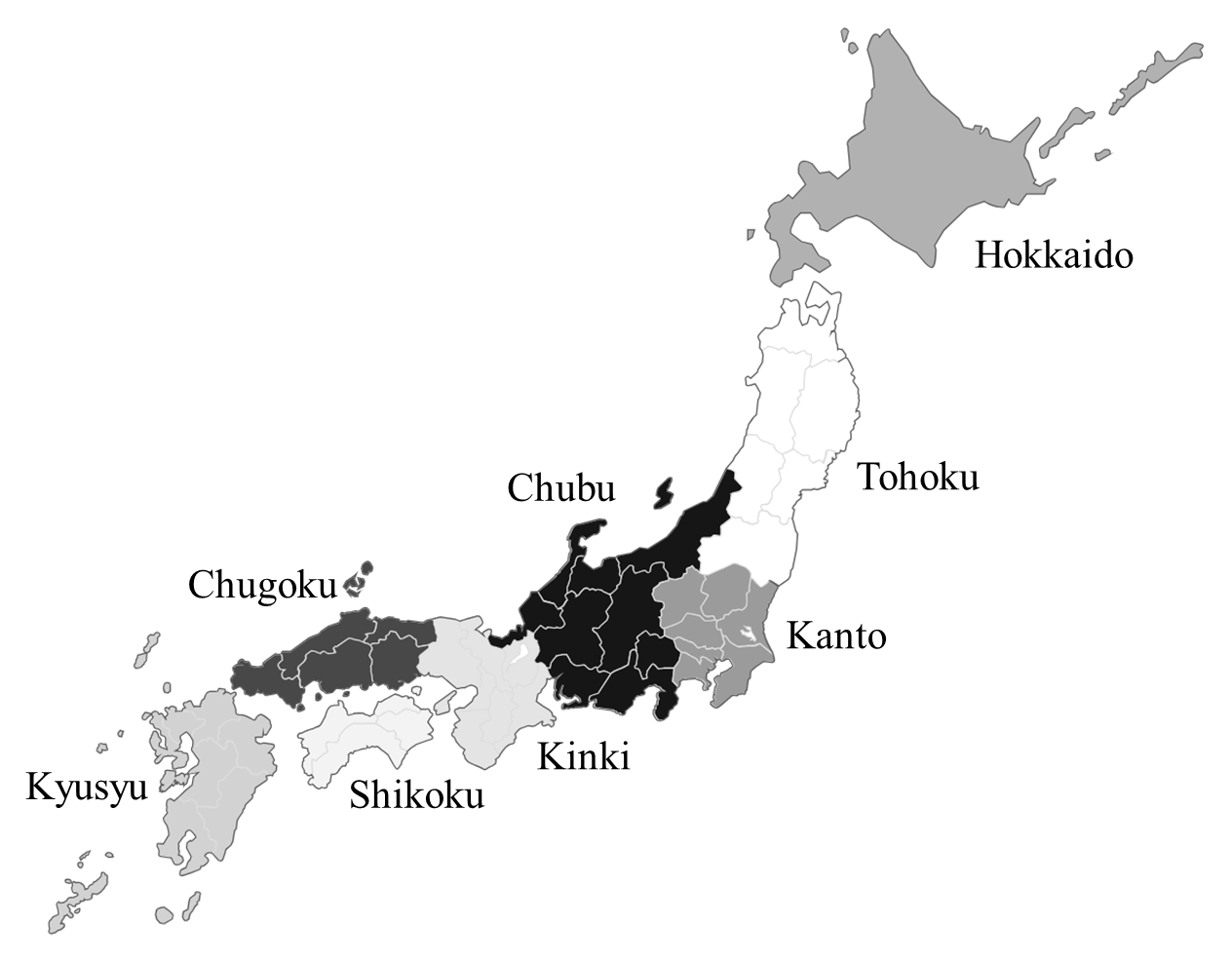
**

This figure was created using Start Point, a web-based platform (https://www.start-point.net/), which allows free use without copyright restrictions.
